# Small-Angle Scattering from Weakly Correlated Nanoscale Mass Fractal Aggregates

**DOI:** 10.3390/nano9040648

**Published:** 2019-04-22

**Authors:** Eugen Mircea Anitas

**Affiliations:** 1 Joint Institute for Nuclear Research, Dubna 141980, Russia; anitas@theor.jinr.ru; 2 Horia Hulubei, National Institute of Physics and Nuclear Engineering, Magurele, Bucharest 077125, Romania

**Keywords:** small-angle scattering, form factor, structure factor, fractals, aggregation

## Abstract

Formation of fractal aggregates is generally an undesired effect which may lead to end products with worse properties as compared to those of the individual components, especially in nanocomposite materials. Although several methods exist to overcome this issue, such as inclusion of additives, irradiation grafting or sonication, their effectiveness relies on a detailed knowledge of the structural properties of the aggregates. Here, small-angle scattering (SAS) technique is used and a theoretical model based on a unified Guinier–Porod approach with weak correlations is developed for investigating the structural properties of nanoscale fractal aggregates. It is shown how one can extract information concerning the correlation length/degree between aggregates, their fractal dimension and the overall size. These parameters can be used for development of various types of novel nanomaterials with pre-determined properties and functions.

## 1. Introduction

Polymer materials reinforced with various kinds of nano-particles or fibers, such as silica, carbon, carbonyl iron or metal oxides results in the formation of nanocomposite materials with improved physical properties [1,2,3,4,5]. This is due to the fact that the composite retains the properties of both the polymer matrix and of the filler. Important parameters affecting their performance are the type, size, volume fraction of the fillers inside the polymer matrix or the interface interaction between fillers and matrix. These parameters have a direct influence on the physical properties of the composites, such as mechanical, electric or dielectric ones [6,7,8,9]. For example, automotive [10], aerospace [11] or sport [12] industries require strong lightweight materials, and therefore the lower the weight of the matrix, the lighter the end product, while the higher the cross-link density, the higher the stiffness and strength. Other applications may require high interfacial areas, which implies reinforcement with very small fillers, enhancing the chemical activity of the fillers surface and so on.

In order to fulfill these requirements and to obtain composite materials with predefined functions and properties, various preparation methods have been developed [13,14]. The most common ones are blending, which involves a mechanical mixing/shearing of the fillers inside the matrix, sol-gel reactions, which involves conversion of monomers into a colloidal solution that acts as a precursor, or in situ polymerization, which involves dispersion of fillers in the monomers, followed by a polymerization. However, especially when the blending (of either melt or solution) methods are used the particles tend to agglomerate. As a consequence, the sought properties can be severely affected, where the end product can have even worse properties than the individual components.

Therefore, in preparation of high-performance composite materials, an effective dispersion is one of the key issues which needs to be controlled. This issue has been partially solved by using various methods, such as addition of additives, irradiation grafting [15] or sonication [16]. However, for an effective application of these methods, and eventually for developing new ones, a qualitative description of the size, shape and nature of the aggregates is required throughout a macroscopic volume of the composite [17,18]. The size of most of these aggregates lie somewhere in-between nano- and micro-scales while their morphology can take very complex shapes, including formation of self-similar hierarchical structures.

Thus, theoretically the proper “language” to describe such complex structures is provided by fractal geometry [19], while small-angle scattering (SAS) of neutrons (SANS), x-rays (SAXS), light (SALS) is often the only method able to provide statistically significant fractal parameters [20,21]. This is due to the fact that a SAS experiment requires almost no a priori preparation and the extracted quantities are averaged over a macroscopic volume.

Here, SAS technique is used to investigate the structural properties of nanoscale fractal aggregates inside composite materials obtained by blending methods. To this aim, we develop a theoretical model based on the unified Guinier–Porod approach [22] together with an interacting factor, and show how it can be used to extract structural information about the size of the aggregate, fractal dimension, pair distribution function and the correlations between aggregates, from SAS data. In order to illustrate the above properties for physical systems, we apply the developed model to a set of data from simulated diffusion limited aggregation (DLA)-based structures at various concentrations.

## 2. Theoretical Background

In a SAS experiment, the differential elastic cross section per unit solid angle (dσ/dΩ) is obtained as a function of the momentum transfer ℏq. This describes, through a Fourier transform, the partial density–density correlations in the sample. The scattering intensity I(q) is usually normalized per unit volume $V^{\prime }$ of the sample, and thus $I\left(q\right)=\left(1/V^{\prime }\right)d\sigma /d\Omega$ [20]. The momentum transfer is related to the scattering angle 2θ through $q=\left(4\pi /\lambda \right)\sin\theta$, where λ is the wavelength of the radiation used. Typically, in a scattering experiment, the structural properties are measured in a range $10^{-3}$ Å${}^{-1}≲q≲1$ Å${}^{-1}$. Taking into account that q=2π/d, where *d* is the maximum size of the investigated object, it turns out that SAS technique can probe structures with dimensions in the range 6 Å÷ 600 Å. However, some extensions up to tens of micrometers are possible by using special instrumental configurations, such as in ultra-SANS/SAXS [23].

If a beam of neutrons, X-ray or light irradiates a volume $V^{\prime }$ of a sample consisting of objects with scattering length $b_{j}$, then by neglecting multiple scattering, the differential cross section of the sample can be written as $d\sigma /d\Omega ={\left|A\left(q\right)\right|}^{2}$, where $A\left(q\right)=\int_{V^{\prime }}\rho_{s}\left(r\right)\exp\left(iq\cdot r\right)d^{3}r$ is the total scattering amplitude [20]. Here, $\rho_{s}\left(r\right)=\sum_{j}b_{j}\delta \left(r-r_{j}\right)$ is the scattering length density (SLD) defined in terms of Dirac’s delta function, and $r_{j}$ are the positions of the scattering objects.

We restrict here to a two-phase system composed of scattering units of SLD $\rho_{p}$ immersed into a matrix with SLD $\rho_{m}$. By subtracting the matrix density from the particle density, we obtain a system in which the particles with scattering contrast $\Delta \rho =\rho_{p}-\rho_{m}$ are fixed in vacuum. Since the total scattering amplitude is the sum of amplitudes of scattering objects, then for monodisperse particles of volume *V* and concentration *n*, the scattering intensity can be written as [20]:

(1) $$I\left(q\right)={n|\Delta \rho |}^{2}P\left(q\right)S\left(q\right),$$

where P(q) is a function of the particle form factor which will be defined below and which describes the structure of the particle, while S(q) is the structure factor which describes the interference scattering from different particles and contains information about their correlations. In the limit of very low concentrations, S(q)=1, and it is usually neglected in Equation (1). P(q) is the square of the modulus of the form factor F(q) averaged over the ensemble of particles, that is $P\left(q\right)=\langle {\left|F\left(q\right)\right|}^{2}\rangle$, where $F\left(q\right)=\left(1/V\right)\int_{V}\exp\left(iq\cdot r\right)d^{3}r$, with F(0)=1. For orientations in 3D space the ensemble averaging is calculated by integrating over the solid angle in spherical coordinates, that is by evaluating [20]:

(2) $$\langle f(q_{x},q_{y},q_{z})\rangle =\frac{1}{4\pi }\int_{0}^{\pi }d\theta \sin\theta \int_{0}^{2\pi }d\theta f\left(q,\theta ,\phi \right),$$

where $q_{x}=q\cos\phi \sin\theta$, $q_{y}=q\sin\phi \sin\theta$ and $q_{z}=q\cos\theta$. The form factor of many basic shapes, as well as for several types of deterministic mass and surface fractals, are known analytically. For a ball of unit radius the form factor is given by [20]:

(3) $$F_{0}\left(q\right)=\frac{3\left(\sin q-q\cos q\right)}{q^{3}}.$$

For mass fractals with a single structural level, variation of density as a function of distance *r* from a reference point inside the aggregate can be described by a pair distribution function of the form [24]:

(4) $$g\left(r\right)\propto r^{D_{m}-3},$$

where $0<D_{m}<3$ is the fractal dimension of the aggregate and quantifies the manner in which the mass *M* of the fractal increase with distance *r*, through $M\propto r^{D_{m}}$. This expression is valid within the fractal region $l_{\min}≲r≲l$ in which one can observe experimentally the fractal properties. Here, *l* is the overall size of the fractal, and $l_{\min}$ is the minimal distance between the scattering units. Since the structure factor S(q) in Equation (1) is proportional to the Fourier transform of the pair distribution function g(r), then by using Equation (4), in the range $1/l≲q≲1/l_{\min}$ one can write [24,25,26]:

(5) $$I\left(q\right)\propto q^{-D_{m}}.$$

In order to describe scattering from complex systems that contains multiple levels of related structural levels, the most widely used model was introduced by Beaucage in Ref. [27], and is based on a generalization of a polymer fractal model. At each structural level the Beaucage model contains a term of the form given by Equation (5). For a single structural level the corresponding scattering intensity is expressed as [27]:

(6) $$I\left(q\right)=G\exp\left(-q^{2}R_{g}^{2}/3\right)+Bq^{-P}{\left[\mathrm{erf}\left(qR_{g}/\sqrt{6}\right)\right]}^{3P},$$

where *G* is the exponential (Guinier) prefactor, $R_{g}$ is the radius of gyration, *B* is a prefactor specific to the type of power-law scattering determined by the regime in which *P* falls, *P* is the Porod exponent, with $P=D_{m}$ for mass fractals, and $\mathrm{erf}\left(\cdot \right)$ is the error function which assures a smooth transition between the Guinier region at low *q* and Porod region at high *q*. In particular, for P=2 we have $B=2G/R_{g}^{2}$, while for an arbitrarily polymeric mass fractal, it has been claimed that [28]:

(7) $$B=\left(GP/R_{g}^{P}\right)\Gamma \left(P/2\right),$$

where $\Gamma \left(\cdot \right)$ is the gamma function. Based on this form, the model has been generalized to describe peaks in SAS data, by including a structure factor of the form [29]:

(8) $$S\left(q\right)=\frac{1}{1+kF_{0}\left(q\xi \right)},$$

where the parameter *k*, with 0<k<6 describes the degree of correlations over a distance ξ [30], and $F_{0}\left(\cdot \right)$ is given by Equation (3). However, for weak correlation we have 0<k<3 [29]. The resulting model assumes symmetric particles with no mutual interaction other than impenetrability. The later condition is always realized for spherical particles, while for not too anisotropic particles and without order at long distances, this serves as a first approximation. Since mass fractal aggregates are more compact with increasing fractal dimension, the higher $D_{m}$ the better the approximation.

Relatively recently it has been shown that in the case of arbitrarily mass fractals, the prefactor *B* in Equation (7) is missing the factor ${\left\{GD_{m}^{2}/\left[\left(2+D_{m}\right)\left(2+2D_{m}\right)\right]\right\}}^{D_{m}/2}$ [31]. Also, the practice of allowing Guinier and Porod factors to vary independently during nonlinear least-squares fitting procedures leads to undesired artefacts, which occur due to relaxation of the vertical linking between Guinier and Porod regions, and which determine the appearance of "kinks" in the scattering curves [31].

As a consequence, an improved model which corrects these issues was proposed in Ref. [22], in which multiple Guinier and Porod regions can be identified. In its simplest form, that is when the scattering objects are spherical, it reads as [22]:

(9) $$P\left(q\right)=\{\begin{array}{ll} G\exp\left(-q^{2}R_{g}^{2}/3\right) & ,\;\;\;q\leq q_{1}\\ Dq^{-D_{m}} & ,\;\;\;q>q_{1}, \end{array}$$

where *D* is a prefactor, and together with the quantity $q_{1}$, are obtained from the continuity conditions of the Guinier and Porod terms as well as of their derivatives. They can be written explicitly as:

(10) $$q_{1}=\left(\frac{1}{R_{g}}\right){\left(\frac{3D_{m}}{2}\right)}^{1/2}$$

and respectively:

(11) $$D=G\exp\left(-q_{1}^{2}R_{g}^{2}/3\right)q_{1}^{D_{m}}.$$

However, this model does not describe SAS data in which peaks and oscillations in power-law decays are present.

## 3. Results and Discussion

### 3.1. A Unified Guinier–Porod Model with Spherical Correlations

**Figure 1:**
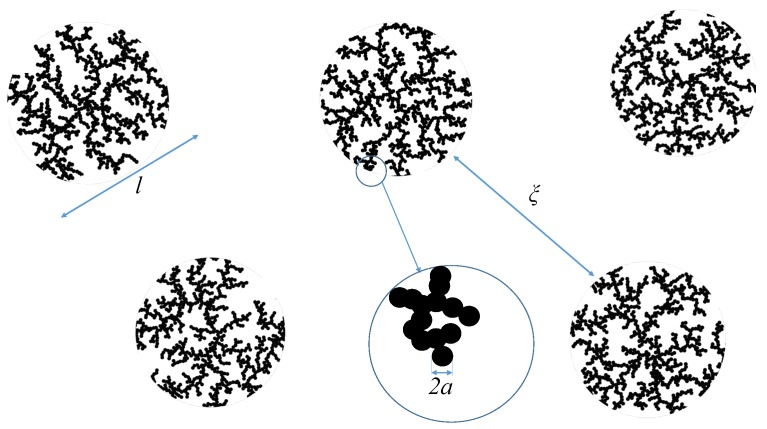
(Color online) Schematic representation of a two-dimensional model of interacting mass-fractal clusters of average overall size *l*, situated at distance ≃ξ apart from each other, and consisting of spherical units of radius *a*, with a≪l.

Here, to avoid the artefacts introduced by using the Beaucage model, as well as for accounting the effects occurring from weakly interacting complex structures, we propose a new approach which takes into account the form factor given by Guinier–Porod model in Equation (9) together with the structure factor of weakly correlated systems given in Equation (8). This can be written as:

(12) $$I\left(q\right)=\{\begin{array}{ll} \frac{G}{1+kF_{0}\left(q\xi \right)}\exp\left(-q^{2}R_{g}^{2}/3\right) & ,\;\;\;q\leq q_{1}\\ \frac{D}{1+kF_{0}\left(q\xi \right)}q^{-D_{m}} & ,\;\;\;q>q_{1}, \end{array}$$

where the fitting parameters are $k,\xi ,R_{g},G$ and $D_{m}$, and were described above. The last equation can approximate situations in which globular-like fractals are distributed in such a way that they have a certain degree of correlations *k* between them over a distance ξ>l (see Figure 1), where *l* is the fractal size. In cases where the high-*q* background level is of interest, a constant term can be added to Equation (12). A generalization to multiple structural levels can be performed by considering that the fractal scattering units (of size *a* in Figure 1) is itself a fractal. As a consequence if $a\ll l_{\min}$, a second Guinier region followed by a Porod one shall be clearly observable at higher values of the momentum transfer.

**Figure 2:**
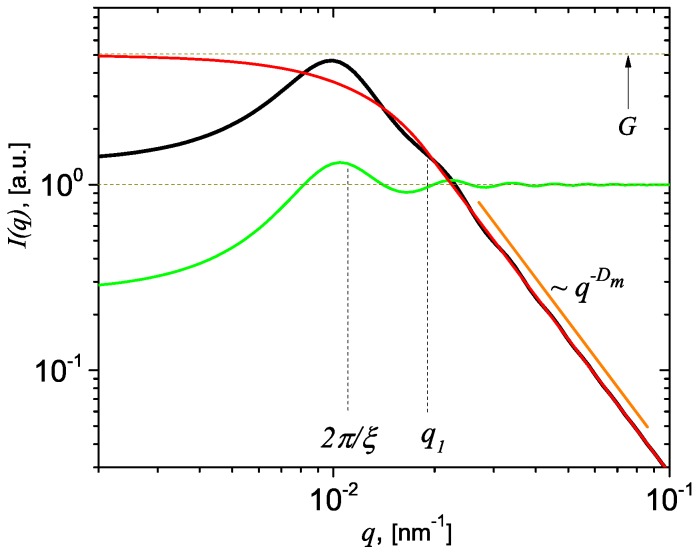
(Color online) Graphical representation of the total scattering intensity I(q) (black) given by model described in Equation (12) (black), the form factor P(q) (red) given by Equation (9) and the structure factor S(q) (green) given by Equation (8) at chosen values of the fitting parameters: $R_{g}=100$ nm, $D_{m}=2.4$, ξ=550 nm, G=5 and k=2.8. Horizontal lines represent the asymptotic values of form factor at low *q* (upper line) and of structure factor at high *q* (lower line). The vertical lines show the position of first maxima in the structure factor (q≃2π/ξ) and of the transition point $q_{1}$ given by Equation (10), between the Guinier and Porod regimes.

The main features of the scattering curve given by Equation (12) are shown in Figure 2 (black) for particular values of the control parameters (see caption of Equation (2) for more details). The curve clearly shows a peak at $q_{\max}≃2\pi /\xi$ followed by small oscillations superimposed on a power-law decay with the scattering exponent equal to the mass fractal dimension $D_{m}$ (i.e., a fractal region). The value of the wavevector at $q≃q_{1}$ indicates the beginning of the fractal region. In order to understand the origin of both the peak and the small oscillations appearing in the fractal region, we need understand first the contribution of each factor in Equation (12), when it is viewed as a product of the form and structure factors given by Equations (9) and (8), according to the decomposition expressed by Equation (1). Thus, Figure 2 shows also the contribution of the form and structure factors (in red, and respectively green) for the same values of the control parameters. The results clearly show that the form factor is proportional to *G* at low *q*, and is followed by a simple power-law decay, i.e., decay without oscillations, at $q≳q_{1}$. However, the structure factor has its most pronounced peak at $q≃q_{\max}$, followed by smaller ones, eventually achieving a complete dampening at high *q*, where S(q)≃1. The height of each peak is related to the parameter *k* in Equation (12). This first peak is enough pronounced, relative to the value of the form factor, such that when multiplied to the form factor, it leads to the appearance of a clear peak in the total intensity. The subsequent peaks in the structure factor have a smaller influence on the total intensity since they are more damped, but still some oscillations are observable (compare red and black curves at $q>q_{1}$). Therefore, the structure factor is responsible for both the peak at $q≃q_{\max}$ and for the small oscillations at $q>q_{1}$, while the form factor is responsible for the power-law decay, also at $q>q_{1}$, in the total scattering intensity. However, depending on the resolution function of the scattering instrument, the small oscillations at high *q* may be completely smeared out.

### 3.2. Application to DLA Fractals

**Figure 3:**
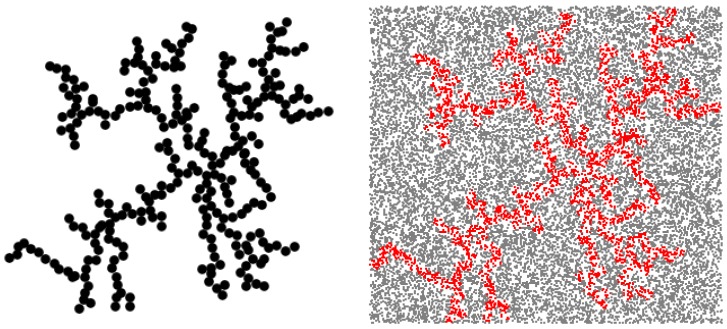
(Color online) **Left-side**: Representation of a 2D DLA with 284 disks as basic units. **Right-side**: Monte Carlos simulation of DLA: Red points are inside the DLA, grey points are outside.

Four SAS data sets have been simulated using Monte Carlo methods applied to 3D diffusion limited aggregation (DLA) clusters (Figure 3 left-side) at various volume fractions: ϕ=0.35 (S1), ϕ=0.36 (S2), ϕ=0.37 (S3), ϕ=0.38 (S4). SAS data have been calculated by first generating a set of random points within the volume of the object, then calculating the corresponding pair distribution function, and finally the scattering intensity I(q) through a Fourier transform [20]. Figure 3 right-side shows a set of 2 × 10${}^{4}$ points generated in a square box delimiting the DLA. The points situated outside DLA (grey) are eliminated and the points inside DLA (red) are used to calculate p(r).

**Figure 4:**
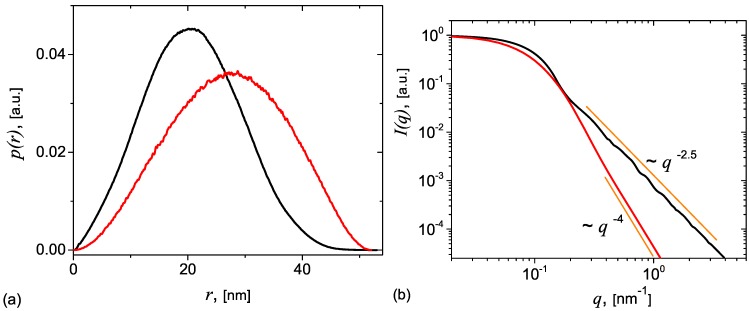
(Color online) (**a**) Pair distribution function p(r) of a 3D diffusion limited aggregation (DLA) cluster (black) and of a sphere of the same size (red). The number of distances used to calculate p(r) for each structure is $≃2.32\times 10^{6}$. (**b**) The corresponding form factors: DLA cluster (black), sphere (red).

The p(r) function for the 3D DLA ($p_{\mathrm{DLA}}\left(r\right)$) is presented in Figure 4a (black) together with that of a sphere ($p_{S}\left(r\right)$) with the same size (red), in order to reflect the degree of asymmetry of the DLA. The results show that both p(r)→0 at r≃52 nm, and which gives the maximum distance ($D_{\max}$) inside these structures. Also, they have a Gaussian-like shape with a full width at half maximum smaller for $p_{\mathrm{DLA}}\left(r\right)$, which arises due to a narrower range of values of distances between arbitrarily points in DLA. The maximum of $p_{\mathrm{DLA}}\left(r\right)$ is situated at higher values of p(r), as compared to those of $p_{S}\left(r\right)$, and indicates a relatively higher number of most common distances inside DLA.

Figure 4 right-side shows the corresponding scattering curves for DLA (black) and sphere (red). The main feature is the presence of the Guinier region at $q≲2\pi /D_{\max}≃0.12$ nm${}^{-1}$, followed by a fractal region with scattering exponent 2.5. Similar values of the fractal dimension of 3D DLA have been reported also in Ref. [32]. Note that a log-normal distribution of the sizes (see details in Ref. [33]) has been considered in calculating I(q).

The structure factor S(q) is simulated through a Percus–Yevick approximation, which allows to obtain an analytic solution of the Ornstein–Zenicke equation, in the form [34]:

(13) $$c\left(r\right)=\{\begin{array}{ll} -\lambda_{1}-6\phi \lambda_{2}r/D_{\max}-\left(\phi /2\right)\lambda_{1}\left(r^{3}/D_{\max}^{3}\right) & ,r\leq D_{\max}\\ 0 & ,r>D_{\max}, \end{array}$$

where $\lambda_{1}={\left(1+2\phi \right)}^{2}/{\left(1-\phi \right)}^{4}$, $\lambda_{2}=-{\left(1+\phi /2\right)}^{2}/{\left(1-\phi \right)}^{4}$, and ϕ is the volume fraction. By performing a Fourier transform of Equation (13) one obtains [34]:

(14) $$S\left(q\right)=\frac{1}{1-M(q)},$$

where $M\left(q\right)=-24\phi \left(\lambda_{1}F_{0}\left(qD_{\max}\right)-6\phi \lambda_{2}T\left(qD_{\max}\right)-\phi \lambda_{1}U\left(qD_{\max}\right)/2\right)$, $F_{0}\left(\cdot \right)$ is the spherical form factor given by Equation (3), while $T\left(q\right)=\left(q^{2}\cos q-2q\sin q-2\cos q+2\right)/q^{4}$, and $U\left(q\right)=\left(q^{4}\cos q-4q^{3}\sin q-12q^{2}\cos q+24q\sin q+24\cos q-24\right)/q^{6}$ [34].

**Figure 5:**
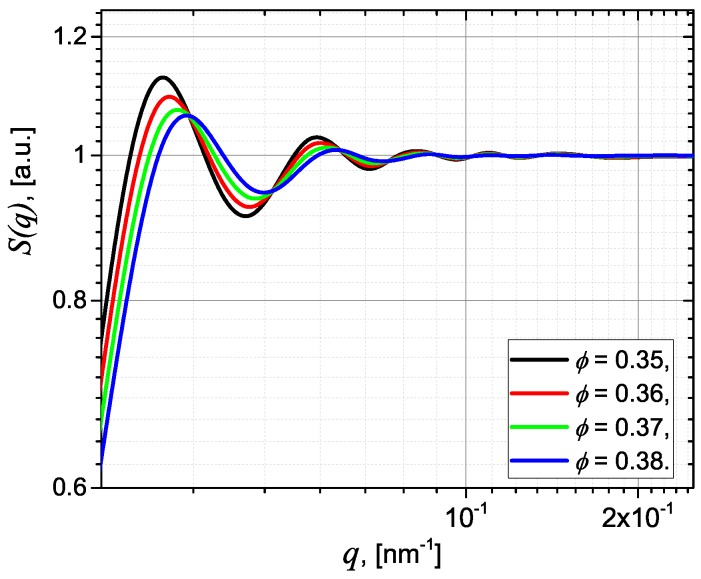
(Color online) Structure factor given by Equation (14) at different values of particle volume fractions ϕ.

**Figure 6:**
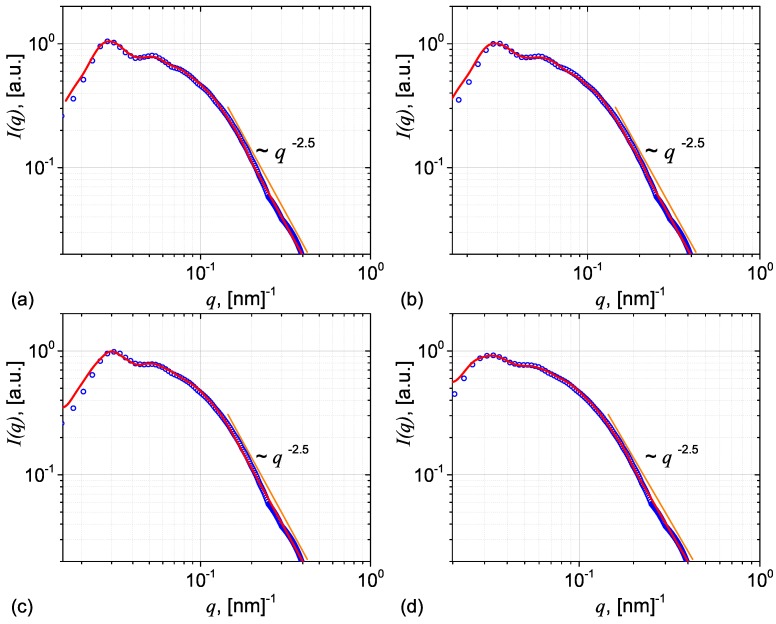
(Color online) Scattering intensities for systems S1 (**a**), S2 (**b**), S3 (**c**) and S4 (**d**). Blue = discrete dots: Monte Carlo simulations; Red = continuous line: Fit with model given by Equation (12).

The corresponding structure factors for S1, S2, S3 and S4 are presented in Figure 5 and show that the amplitudes of the oscillations decrease with increasing ϕ from 0.35 to 0.38. The scattering intensity, which involves the product of the form factor in Figure 4b and the structure factors in Figure 5 is shown in Figure 6 (blue = discrete points). The continuous (red) lines are fits using Equation (12), where ξ=197 nm, and k≃2.91 (S1), k≃2.46 (S2), k≃2.08 (S3), and k≃1.52 (S4), indicating a decrease of the correlation degree with the volume fraction. Note that all scattering curves are characterized by the presence of small oscillations at small *q*, followed by a power-law decay with scattering exponent 2.5, as expected. Such a behaviour is often seen in polymer nanocomposites where fractal aggregates are formed in the matrix, such as in situ filled siloxane rubber with a Polydimethylsiloxane (PDMS) precursor [29] or in silica nanoparticles embedded in an epoxy resin matrix [35,36].

## 4. Conclusions

We introduce a model that can be used to analyze SAS data from interacting mass fractal aggregates with a single structural level. The model is based on the unified Guinier–Porod form factor together with a structure factor which considers weakly interacting systems between aggregates. By using this approach we are able to quantify the degree of correlations and to extract the main structural properties of the fractal aggregates. In order to illustrate the above findings we apply the developed model to a set of data from DLA-based aggregates at various concentrations and determine the correlation length, fractal dimension and radius of gyration of the aggregates as well as the pair distribution function of the aggregate.

The model can be generalized to describe experimental SAS data showing two Guinier regions or a superposition of maxima and minima in the fractal region. This can be usefull in extracting additional structural information about aggregates consisting of multiple structural levels arising from various nano-scale processes.
